# Glycine alleviated diquat-induced hepatic injury via inhibiting ferroptosis in weaned piglets

**DOI:** 10.5713/ab.21.0298

**Published:** 2022-01-03

**Authors:** Hongwei Hua, Xiao Xu, Wei Tian, Pei Li, Huiling Zhu, Wenjun Wang, Yulan Liu, Kan Xiao

**Affiliations:** 1 Hubei Key Laboratory of Animal Nutrition and Feed Science, Wuhan Polytechnic University, Wuhan 430023, China; 2 College of Life Science, South-Central University for Nationalities, Wuhan 430074, China

**Keywords:** Ferroptosis, Glycine, Liver, Oxidative Stress, Weaned Piglets

## Abstract

**Objective:**

The beneficial effects of glycine were tested in piglets with diquat-induced hepatic injury.

**Methods:**

Thirty-two piglets were assigned by a 2×2 factorial experimental design including glycine supplementation and diquat challenge. After 3 weeks of feeding with a basic diet or a 1% glycine supplemented diet, piglets were challenged with diquat or saline. After 1 week later, the piglets were slaughtered and samples were collected.

**Results:**

Our results indicated that glycine alleviated diquat induced morphological hepatic injury, decreased the activities of plasma alanine aminotransferase, aspartate aminotransferase and glutamyl transpeptidase in the piglets under diquat challenge, and increased total antioxidant capacity and antioxidative enzyme activity significantly. Adding glycine enhanced the concentrations of hepatic adenosine triphosphate and adenosine diphosphate. Transmission electron microscope observation showed that diquat induced clear hepatocytes ferroptosis and its effect could be alleviated by glycine to a certain degree. Moreover, glycine significantly affected mRNA and protein expression of ferroptosis-related signals in the liver.

**Conclusion:**

These results demonstrated that glycine attenuated liver damage via inhibiting ferroptosis.

## INTRODUCTION

The liver, as one of the largest internal organs in our body, is involved in several essential physiological metabolic processes, such as detoxification, drug metabolism and synthesis of liver glycogen [1]. The status of the liver is an important criterion to evaluate the health level of the body. A wide range of factors can lead to liver diseases [2,3]. Meanwhile, oxidative stress has been recognized as a fundamental factor in various liver diseases [4–6]. Usually, the body is under a balance between pro-oxidant and anti-oxidant defense by internal sophisticated anti-oxidant system. However, when the balance is broken, the body will be under a situation called oxidative stress [7].

Reactive oxygen species (ROS) are a key intermediate product in oxidative stress process, and can interfere in electron transport in the mitochondrial membrane and eventually cause membrane lipid peroxidation [8–10]. Lipoperoxidation was considered as the most significant reason of cell damage and death [11]. In other words, the oxidative stress process can easily cause cell death, and then induce tissue impairment and endanger health. In 2012, Dixon et al [12] found that there was an original and nonapoptotic cell death form which was closely related to the process of oxidative stress, named ferroptosis. Ferroptosis, as a kind of programmed cell death, relied on iron gathering and originated by the malfunction of the glutathione (GSH)-dependent antioxidant defenses, resulting in unchecked lipid peroxidation and eventual cell death [12]. Ferroptosis was initiated by iron overload and regulated by transferrin receptor protein (TFR1)/Fe2^+^ and System Xc-/glutathione peroxidase 4 (GPX4) signaling pathway [12]. One the ferroptosis occurred, the mitochondria would be damaged and dysfunction, which lead to the dysregulation of energy metabolism.

Traditionally, glycine is considered to be a nutritionally nonessential amino acid as it can be synthesized in the body [13]. But ample evidences have shown that the synthesized de novo glycine cannot meet neonatal growth and development of piglets [14–16]. Recently, more and more reports declared that glycine played important roles in alleviating oxidative stress and liver injury [17–19]. Glycine is a component of the antioxidant glutathione peroxidase (GSH-PX). Meanwhile, GPX4 is an important regulator in the process of ferroptosis [20]. Thus, glycine might have the potential to alleviate ferroptosis.

Based on the foregoing, it was hypothesized that supplementation with glycine could alleviate hepatic injury by regulating the degree of oxidative stress and ferroptosis. In the present trial, diquat was intraperitoneally injected at 10 mg/kg body weight (BW) to establish the oxidative stress model. Injection with diquat is a common method inducing oxidative stress in piglets [21]. It has been reported that diquat caused hepatocyte caryolysis, karyopycnosis, and altered hepatic cord arrangement in the piglets [21]. This study aimed to explore whether glycine could alleviate diquat-induced liver damage in piglets and the correlation between ferroptosis and hepatocyte death induced by diquat.

## MATERIALS AND METHODS

### Animal care and experimental design

Animal experimental procedures were approved by the Animal Care and Use Committee of Wuhan Polytechnic University (Wuhan, China). The Approval Number of IACUC was EM678 (November 18, 2018). Thirty-two crossbred piglets (Duroc×Large White×Landrace, initial BW 7.18±0.70 kg, weaned at 21±1 d) were randomly allocated by weight to 4 groups with 8 replicate pens for each group. The piglets were housed individually (1.80×1.10 m^2^) in pens and allowed *ad libitum* access to feed and water. The basic diets were formulated according to the nutrient requirements of the NRC (2012) [22]. Lighting was natural and room temperature was maintained at 22°C to 25°C. Piglets were fed basic diet or glycine supplementation diet for 21 d before diquat challenge. The experiment was conducted by a 2×2 factorial design. The main factors were glycine (piglets fed either basic diet or 1% glycine supplementation diet) and diquat challenge (piglets treated with diquat or sterile saline). On d 21, the piglets in challenge groups were IP injected with diquat (dibromide monohydrate, Chem Service, West Chester, PA, USA) at 10 mg/kg BW and the piglets in unchallenged groups were injected with the equivalent amount of saline. The dose of glycine supplementation was chosen according to Wu et al [23] and our preliminary research, and dose of diquat was chosen according to Lv at al [21] and Cao et al [24].

### Blood and liver sample collection

One week after injection of diquat or saline, blood samples were collected from jugular vein and centrifuged to harvest plasma. Then all piglets were slaughtered using sodium pentobarbital (80 mg/kg BW) and the liver samples were collected instantly. One fragment of liver sample was stored in fresh 4% paraformaldehyde/phosphate buffered saline at least for 24 h for histological analysis. The remaining portions were immediately frozen in liquid nitrogen, and then stored at −80°C for further analysis.

### Measurement of plasma biochemical parameters

Plasma aspartate aminotransferase (AST), alanine aminotransferase (ALT), and glutamyl transpeptidase (GGT) activities were determined referred to our previous study [2].

### Liver morphology analysis

After a 24 h fixation, the liver segments (5 mm) for liver morphology were deparaffinized and stained with hematoxylin and eosin. Morphology analysis was performed according to our previous study [2].

### ATP, ADP, and AMP concentrations measurement

The hepatic concentrations of adenosine triphosphate (ATP), adenosine diphosphate (ADP), and adenosine monophosphate (AMP) were analyzed with high performance liquid chromatography referred to previous study [25].

### Hepatic anti-oxidative capacity measurement

The total antioxidative capacity (TAOC), content of malondialdehyde (MDA), activities of GSH-PX and content of reduced GSH of liver samples were determined using commercially available kits (Nanjing Jiancheng Bioengineering Co. Ltd., Nanjing, China). All indexes were measured referred to the protocols of the manufacturers.

### Transmission electron microscope observation of the liver

The hepatocytes ultrastructure were observed and photographed with a HT7700 TEM (Hitachi Co. Ltd., Tokyo, Japan) referred to previous study [26].

### mRNA expression analysis by real-time polymerase chain reaction

The methods for total RNA isolation, quantification, reverse transcription, and real-time polymerase chain reaction were the same as our previous study [27]. The primer pairs for amplification of target genes are presented in (Table 1). The expression of the target genes relative to housekeeping gene (glyceraldehyde-3-phosphate dehydrogenase [*GAPDH*]) was analyzed by the 2–ΔΔCT method [28]. Relative mRNA abundance of each target gene was normalized to the piglets fed basic diet and injected with saline.

### Protein abundance analysis by Western blot

The methods for protein abundance analysis in liver were according to previous research [2]. Specific primary antibodies included rabbit anti-TFR1, goat anti-solute carrier family 7 member 11 (SLC7A11), rabbit anti-GPX4 and mouse anti-β-actin antibody. The relative protein abundance of target proteins (TFR1, SLC7A11, GPX4) were expressed as the ratio of target protein/β-actin protein.

### Statistical analyses

All data were analyzed using the mixed procedure of SAS appropriate for a 2×2 factorial design (SAS Institute, Cary, NC, USA). The statistical model included the effects of diquat challenge, glycine supplementation and their interactions. All data are shown as means±pooled standard error of mean. If there was significant or a trend interaction between diquat and glycine, post hoc testing was conducted by Duncan’s multiple comparison test. Differences were considered statistically significant at p≤0.05 as statistically significant, and 0.05<p≤0.10 as trends.

## RESULTS

### Liver histology

**Figure 1:**
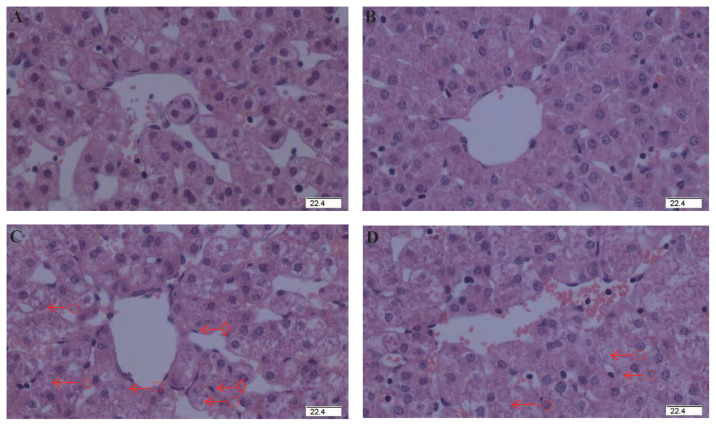
Effect of dietary glycine on hepatic morphology after diquat challenge in weanling piglets. Pigs were firstly feed with a basic diet or a 1% glycine supplemented diet for 3 weeks, and then challenged with diquat or saline. Representative hepatic photomicrographs were shown. (A) Pigs receiving a control diet and injected with saline. (B) Pigs receiving a glycine supplementation diet and injected with saline. No obvious pathological changes were observed. (C) Pigs receiving the same control diet and injected with diquat. Significant pathological changes of hepatic injury such as hepatic spindle cells disappear (○), heptatocyte caryolysis (□), heptatocyte karyopycnosis (⋄), hepatic cell cords arrangement in disorder were found. (D) Pigs receiving a glycine supplementation diet and injected with diquat. Hepatic injury was significantly relieved. n = 8 (1 piglet per pen). Original magnifications 400×. Scale bars = 22.4 μm.

To explore the effects of glycine on the hepatic injury initiated by diquat, we observed liver tissue sections. No significant pathological changes were observed in the piglets receiving control diet (Figure 1A) or glycine diet (Figure 1B). But some pathological changes of hepatic injury such as hepatocyte caryolysis, karyopycnosis, hepatic spindle cell disappearance and hepatic cell cords arranged in disorder were observed in the piglets injected by diquat and receiving control diet (Figure 1C). Compared with the piglets injected with diquat and receiving control diet, hepatic injury was alleviated in the piglets injected with diquat and receiving glycine diet (Figure 1D). These results demonstrated that glycine diet alleviated hepatic injury induced by diquat challenge in piglets.

### Plasma biochemical parameters

AST, ALT, and GGT are important transaminases mainly existing in liver cells. When the liver is damaged, they are released into the blood. So we measured the plasma level of AST, ALT, and GGT to reflect liver’s function. A significant interaction existed between diquat challenge and dietary glycine for the activities of AST, ALT, and GGT (p<0.05, Table 2). Compared with the piglets injected with saline, the piglets injected with diquat had increased plasma GGT activity (p<0.05). Compared with the piglets received control diet, the piglets received glycine supplementation diet had decreased plasma GGT activity (p<0.05). Supplementation of glycine significantly reduced the activities of AST, ALT, and GGT in plasma of the piglets under the diquat challenge (p<0.05). Those results indicated that glycine supplementation protected the liver’s function when challenged by diquat.

### Concentrations of ATP, ADP, and AMP in the liver

To explore the effects of glycine on the energy metabolism in the liver, we measured the concentration of ATP, ADP, and AMP. As shown in Table 3, a significant interaction existed between diquat challenge and dietary glycine for liver ATP and ADP concentrations (p<0.05). Overall, diquat treatment decreased liver ATP and ADP concentrations (p<0.05) compared with saline treatment. Relative to the piglets fed control diet, the piglets fed glycine supplementation diet had increased ADP (p<0.05) and ATP concentrations (p = 0.082). Supplementation of glycine significantly enhanced liver ATP concentration in the piglets under the diquat challenge (p<0.05). These results suggested the glycine supplementation improved the liver’s energy metabolism after diquat challenge.

### Liver antioxidative capacity

To explore the effects of glycine on the antioxidative capacity in liver, we also measured the TAOC, GSH-PX, and GSH activities and MDA concentration. A significant interaction existed between diquat challenge and dietary glycine for liver TAOC, GSH-PX, and GSH activities and MDA concentration (p<0.05, Table 4). Compared with the piglets injected with saline, the piglets injected with diquat had increased liver MDA amount (p<0.05) and decreased liver GSH-PX and GSH activities (p<0.05). Compared with the piglets received control diet, the piglets received glycine supplementation diet had enhanced liver TAOC, GSH-PX, and GSH activities (p<0.05). Glycine significantly increased liver TAOC, and decrease liver MDA amounts in the piglets under the diquat challenge (p<0.05). Our results demonstrated that glycine improved the antioxidative capacity after diquat challenge in the liver.

### Hepatocytes transmission electron microscope observation

**Figure 2:**
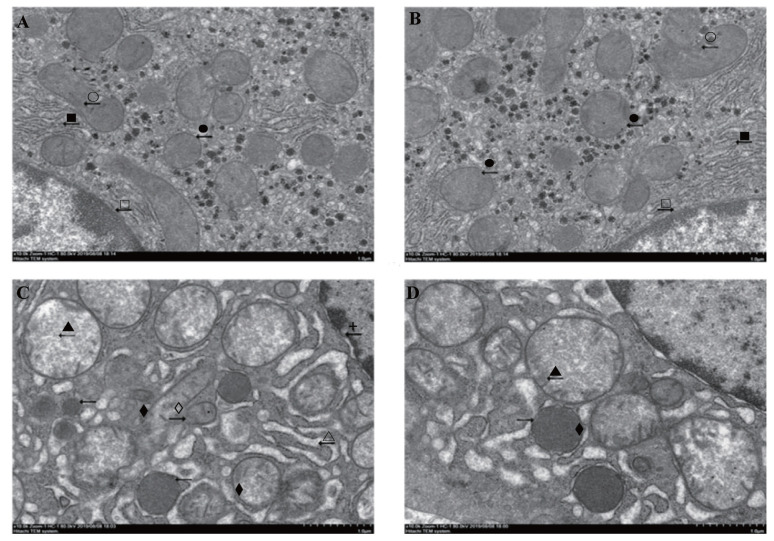
Effect of dietary glycine on hepatocyte ultrastructure after diquat challenge in weanling piglets. Representative hepatocytes ultrastructure photomicrographs were shown. Pigs were firstly feed with a basic diet or a 1% glycine supplemented diet for 3 weeks, and then challenged with diquat or saline. (A) Pigs receiving a control diet and injected with saline. (B) Pigs receiving dietary glycine and injected with saline. (A) and (B) had no obvious ferroptosis features. Presented as complete mitochondria (●), mitochondria with distinct cristae (○), normal rough endoplasmic reticulum (■), karyotheca integrity (□). (C) Pigs receiving the same control diet and injected with diquat. Significant ferroptosis features were observed, such as mitochondrial pyknosis (◆), mitochondrial outer membrane rupture (⋄), mitochondrial cristae reduction (▲), dilatations of rough endoplasmic reticulum (Δ) and karyotheca deformation separated from cytoplasm (+) were observed. (D) Pigs receiving dietary glycine and injected with diquat.

To observe the effects of glycine on hepatocytes ferroptosis in the piglets, we used the transmission electron microscope (TEM) to observe the changes of liver’s characteristics. No obvious characteristics of hepatocytes ferroptosis in the piglets injected with saline and fed control (Figure 2A) or glycine (Figure 2B) diet. Nevertheless, characteristics of ferroptosis such as mitochondrial pyknosis, mitochondrial cristae reduction, mitochondrial outer membrane rupture, and karyotheca deformation separated from cytoplasm were observed in the piglets injected with diquat and receiving control diet (Figure 2C). Relative to the piglets injected with diquat and receiving control diet, hepatocytes ferroptosis was alleviated in the piglets injected with diquat and receiving glycine diet (Figure 2D). Our results indicated that glycine could alleviate the hepatocytes ferroptosis in the piglets.

### Liver mRNA expressions of the key factors in ferroptosis

To explore the effects of glycine on the ferroptosis signaling pathway, we measured mRNA abundance of the key factors in the signal. Overall, a significant interaction existed between diquat injection and dietary glycine for live TFR1, heat shock protein beta 1 (HSPB1) and SLC7A11 mRNA abundance (p<0.05, Table 5). Relative to the piglets injected with saline, the piglets injected with diquat had increased TFR1 and GPX4 mRNA abundance (p<0.05) in liver. There was no diquat×glycine interaction observed for GPX4 in which glycine increased the GPX4 mRNA expression (p< 0.01) in the presence or absence diquat challenge. Compared with the piglets fed control diet, the piglets fed glycine supplementation diet had enhanced GPX4 mRNA abundance (p<0.05). The supplementation of glycine significantly decreased liver TFR1 mRNA abundance under the diquat challenge (p<0.05). HSPB1 and SLC7A11 mRNA abundance in the piglets injected with diquat and receiving glycine supplementation diet were higher than the diquat challenged piglets fed control diet (p<0.05). Our results demonstrated that glycine supplementation alleviated the mRNA expressions of the key factors related in ferroptosis signaling pathway.

### Liver protein abundance in ferroptosis-related signals

**Figure 3:**
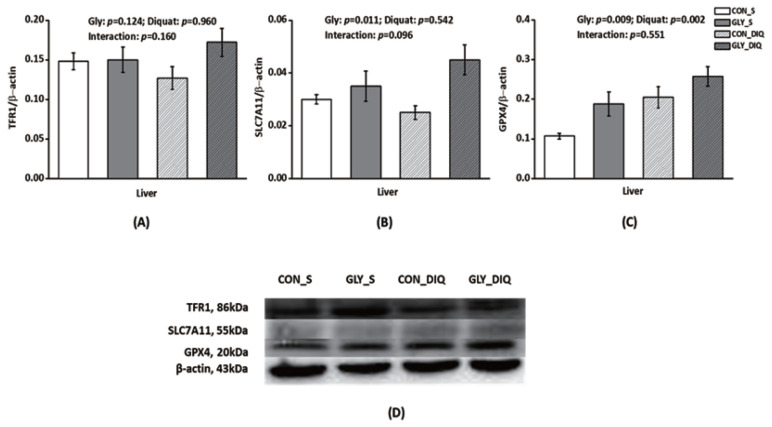
Effect of dietary glycine on liver ferroptosis signals after diquat challenge in weaned piglets. Pigs were firstly feed with a basic diet or a 1% glycine supplemented diet for 3 weeks, and then challenged with diquat or saline. The bands were the representative Western blot images (D). Values were mean and pooled standard error of mean, n = 8 (1 piglet per pen). CON_S, piglets receiving the control diet and injected with saline; GLY_S, piglets fed dietary glycine and injected with saline; CON_DIQ, piglets fed the control diet and injected with diquat; GLY_DIQ, piglets fed dietary glycine and injected with diquat. TFR1, transferrin receptor protein; SLC7A11, solute carrier family 7 member 11; GPX4, glutathione peroxidase 4.

To further explore the effects of glycine on ferroptosis signaling pathway, we measured protein expression of the key factors in the signal. There were no significant interaction existed between diquat injection and dietary glycine for TFR1, SLC7A11, and GPX4 protein abundance in liver (Figure 3). Piglets challenged with diquat had higher liver GPX4 protein abundance compared with the piglets injected with saline (p<0.05). Compared with the piglets received control diet, the piglets received glycine diet had increased GPX4 and SLC7A11 protein abundance in liver (p<0.05). In conclusion, glycine supplementation alleviated the protein expression of ferroptosis signaling pathway in the piglets challenged by diquat.

## DISCUSSION

ALT, AST, and GGT are important transaminases mainly existing in liver cells under normal physiological conditions, which are involved in the bodies’ metabolic process. ALT catalyzes the transamination between glutamate and pyruvate, and AST catalyzes the transamination between glutamate and oxaloacetate. They are regarded as the most sensitive indicators to measure liver function and reflect liver damage. High levels of liver enzymes GGT, ALT, and AST are predictive of disease and all-cause mortality and can reflect liver injury, fatty liver and/or oxidative stress [29]. Our results demonstrated that diquat challenge increased the serum GGT, ALT, and AST, showing destructive effects on liver function. However, in the present study, glycine supplementation reduced plasma ALT, AST, and GGT activities in the piglets injected with diquat, which is in line with the histological observations. The above results indicated that glycine attenuated diquat-induced injury in liver histology and function. Consistent with these findings, previous papers showed that dietary glycine alleviated abnormal levels of serum aminotransferase activity and pathological changes of histopathologic structure of liver tissue in a mouse model, and in a rat model of oxidative stress in the liver [30,31].

Adenosine triphosphate takes part in various cellular functions as well as affords energy [32]. AMP is a good indicator of cellular stress because an increased rate of ATP hydrolysis leads to a rapid accumulation of AMP in the cell [33]. ATP hydrolysis can increase the cellular ADP content, which is converted by adenylate kinase (2ADP↔ATP+AMP) to ATP and AMP [34]. In addition, several reports have indicated that many amino acids such as proline, arginine, and aspartate can be used as an energy source by the body [35–37]. However, there are few reports which showed the effect of glycine on the energy metabolism in the liver of piglets. In the current experiment, glycine supplementation increased the hepatic ATP concentration in the piglets challenged with diquat, which indicated that dietary glycine might mitigate liver injury.

TAOC can reflect cumulative effect of all antioxidants in the organ or body and MDA is a key indicator for oxidative stress [38]. The MDA content is an important parameter that reflects the body’s anti-oxidation potential. It can reflect the body’s lipid peroxidation rate and intensity, and in other words it can indirectly reflect the degree of tissue peroxidation damage. The GSH is a tripeptide consisted with glutamate, cysteine and glycine. Moreover, GSH is considered as a cofactor of GSH-PX which participated in neutralizing H_2_O_2_ and lipid hydroperoxides [39]. In the present experiment, diquat challenge decreased the GSH-PX and GSH contents, and improved the MDA concentration, which suggested an oxidative damage in liver tissue. However, glycine supplementation alleviated diquat-induced oxidative stress in liver via enhancing TAOC, GSH-PX, and GSH levels and restraining MDA generation, indicating dietary glycine may contribute to GSH production and enhance antioxidant capacity of liver. El-Hafidi et al [39] found an improving GSH biosynthesis and protection effects from oxidative stress in the liver through glycine supplementation. Wang et al [40] demonstrated that glycine had a protective effect against oxidative stress in intestinal epithelial cells of pigs.

The classical programmed cell death such as apoptosis and autophagy is considered to be death of a cell in any pathological format when mediated via an intracellular program [41]. Dixon et al [12] found an original and nonapoptotic cell death type related to oxidative stress, named ferroptosis. It was morphologically, biochemically, and genetically different from other classical programmed cell deaths. Ferroptosis was a kind of programmed cell death dependent on iron, and originated by the malfunction of the GSH-dependent antioxidant defenses, causing unchecked lipid peroxidation and eventual cell death [42]. The typical characteristics of ferroptosis were lipid peroxidation, mitochondrial pyknosis, mitochondrial outer membrane rupture and mitochondrial cristae reduction [12]. In our study, the ultrastructure observation displayed clearly feature of ferroptosis in hepatocytes of the piglets injected with diquat, which demonstrated diquat induced hepatic ferroptosis. Furthermore, dietary glycine relieved hepatic ferroptosis in morphology of piglets.

In order to investigate the mechanism of the impact, we measured the mRNA and protein abundance of the signals associated with ferroptosis in liver. TFR1, also named CD71, is a receptor protein encoded by the transferrin receptor gene [43]. This protein can transfer ferric ion into inner membrane when ferroptosis occurs. HSPB1 is a chaperone of the small heat shock protein which participates in inhibition of apoptosis, regulation of cell development, as well as cell differentiation. HSPB1 can decrease the ferric ion concentrations by inhibiting the expression of TFR1, and further alleviate the degree of ferroptosis [44]. *SLC7A11* gene codes for a sodium-independent cystine-glutamate antiporter which is chloride dependent, known as system Xc- or xCT. SLC7A11 it plays a key function in GSH homeostasis which protects cells from oxidative stress [45]. GPX4 can protect cells against membrane lipid peroxidation, and its activity is involved in the capacity of inhibiting ferroptosis [46]. In our study, the enhanced gene expression of TFR1 and GPX4 in the piglets injected with diquat indicated that diquat induced mass ferric ion into hepatocytes which caused hepatic oxidative stress. Diquat challenge activated the ferroptosis signaling pathway and liver’s antioxidant capacity. Simultaneously in order to alleviate oxidative stress, GPX4 activity was enhanced. In addition, dietary glycine increased gene expressions of liver HSPB1, SLC7A11, and GPX4 in the piglets challenged with diquat. These results illustrated that glycine had a positive effect on inhibiting ferroptosis via enhancing liver antioxidant system which was in accordance with the results of liver antioxidant capacity. Furthermore, Ingold et al. found that the utilization of selenium by GPX4 was necessary to prevent hydroperoxide-induced ferroptosis [47]. Similar to the results of gene expression, the protein abundance results also showed that dietary glycine enhanced GPX4 and SLC7A11 abundance, which illustrated that dietary glycine was conducive to protect the liver from ferroptosis via enhancing the synthesis of GPX4.

In conclusion, dietary glycine alleviated diquat-induced hepatic oxidative stress in piglets. Glycine was able to inhibit diquat-induced hepatic ferroptosis via enhancing GPX4 expression.

## Figures and Tables

**Table 1:** Primer sequences used for real-time polymerase chain reaction

Gene	Forward (5′ → 3′)	Reverse (5′→ 3′)
*TFR1*	CGAAGTGGCTGGTCATCT	TGTCTCTTGTCTCTACATTCCT
*HSPB1*	CTCGGAGATCCAGCAGACT	TCGTGCTTGCCCGTGAT
*SLC7A11*	GCCTTGTCCTATGCTGAGTTG	GTTCCAGAATGTAGCGTCCAA
*GPX4*	CTGTTCCGCCTGCTGAA	ACCTCCGTCTTGCCTCAT
*GAPDH*	CGTCCCTGAGACACGATGGT	GCCTTGACTGTGCCGTGGAAT

*TFR1*, transferrin receptor protein 1; *HSPB1*, heat shock protein beta 1; *SLC7A11*, solute carrier family 7 member 11; *GPX4*, glutathione peroxidase 4; *GAPDH*, glyceraldehyde-3-phosphate dehydrogenase.

**Table 2:** The plasma biochemical parameters of the piglets fed the glycine diets with diquat challenge

Item	Saline		Diquat		SEM	p-value		
		
	Control	Glycine	Control	Glycine		Glycine	Diquat	Interaction
AST (U/L)	40.0^b^	40.5^b^	62.1^a^	42.0^b^	6.2	0.242	0.168	0.004
ALT (U/L)	61.1^b^	63.0^b^	72.5^a^	65.3^b^	6.6	0.455	0.163	0.036
GGT (U/L)	40.1^b^	30.4^c^	59.5^a^	33.9^bc^	4.6	0.027	0.015	0.038

Values are mean and pooled SEM, n = 8 (1 pig/pen).

SEM, standard error of mean; AST, aspartate aminotransferase; ALT, alanine aminotransferase; GGT, glutamyl transpeptidase.

a–c Means in a row without a common letter differ, p<0.05.

**Table 3:** The liver ATP, AMP, and ADP contents of the piglets fed the glycine diets with diquat challenge

Item	Saline		Diquat		SEM	p-value		
		
	Control	Glycine	Control	Glycine		Glycine	Diquat	Interaction
ATP (μg/g wet wt)	633^a^	646^a^	581^b^	629^a^	24	0.082	0.018	0.035
ADP (μg/g wet wt)	118^b^	139^a^	103^b^	112^b^	9	0.013	0.015	0.043
AMP (μg/g wet wt)	128	131	127	137	69	0.488	0.788	0.722
TAN^1)^ (μg/g wet wt)	879^a^	916^a^	811^b^	878^a^	28	0.012	0.026	0.018
AEC	0.785	0.78	0.78	0.781	0.008	0.843	0.589	0.537
AMP/ATP	0.202	0.203	0.219	0.218	0.013	0.968	0.329	0.856

Values are mean and pooled SEM, n = 8 (1 pig/pen).

ATP, adenosine triphosphate; AMP, adenosine monophosphate; ADP, adenosine diphosphate; SEM, standard error of mean; TAN, total adenine nucleotides; AEC, adenylate energy content.

a,b Means in a row without a common letter differ, p<0.05.

1) TAN = ATP+ADP+AMP; AEC = (ATP+0.5 ADP)/(ATP+ADP+AMP).

**Table 4:** The liver antioxidative capacity of the piglets fed the glycine diets with diquat challenge

Item	Saline		Diquat		SEM	p-value		
		
	Control	Glycine	Control	Glycine		Glycine	Diquat	Interaction
TAOC (U/mgprot)	3.01^ab^	3.18^a^	2.56^b^	3.18^a^	0.3	0.026	0.127	0.019
GSH-PX (U/mgprot)	177^ab^	202^a^	124^b^	187^ab^	31	0.013	0.032	0.038
GSH (mgGSH/gprot)	60.6^ab^	68.1^a^	46.8^b^	60.6^ab^	9.5	0.021	0.024	0.005
MDA (nmol/mgprot)	3.25^b^	1.85^b^	5.83^a^	3.47^b^	1.00	0.091	<0.001	0.001

Values are mean and pooled SEM, n = 8 (1 pig/pen).

SEM, standard error of mean; TAOC, total antioxidative capacity; GSHPX, glutathione peroxidases; GSH, reduced glutathione; MDA, malondialdehyde.

a,b Means in a row without a common letter differ, p<0.05.

**Table 5:** The liver mRNA expression of ferroptosis-related signals of the piglets fed the glycine diets with diquat challenge

Item	Saline		Diquat		SEM	p-value		
		
	Control	Glycine	Control	Glycine		Glycine	Diquat	Interaction
*TFR1*	1.00^c^	1.05^bc^	1.72^a^	1.32^b^	0.16	0.126	<0.001	0.002
*HSPB1*	1.00^ab^	0.95^b^	0.88^b^	1.17^a^	0.09	0.364	0.242	0.012
*SLC7A11*	1.00^b^	0.93^b^	0.75^b^	1.63^a^	0.19	0.207	0.214	<0.001
*GPX4*	1.00^b^	2.30^a^	1.19^b^	2.54^a^	0.30	<0.001	0.012	0.266

Values are mean and pooled SEM, n = 8 (1 pig/pen).

SEM, standard error of mean; *TFR1*, transferrin receptor protein 1; *HSPB1*, heat shock protein beta 1; *SLC7A11*, solute carrier family 7 member 11; *GPX4*, glutathione peroxidase 4.

a–c Means in a row without a common letter differ, p<0.05.
